# Progress and development on biological information of crop phenotype research applied to real-time variable-rate fertilization

**DOI:** 10.1186/s13007-020-0559-9

**Published:** 2020-02-03

**Authors:** Yinyan Shi, Yang Zhu, Xiaochan Wang, Xin Sun, Yangfen Ding, Wexing Cao, Zhichao Hu

**Affiliations:** 1 Nanjing Research Institute for Agricultural Mechanization, Ministry of Agriculture and Rural Affairs, Nanjing, 210014 China; 2 College of Engineering, Nanjing Agricultural University, Nanjing, 210031 China; 3 Department of Agricultural and Biosystems Engineering, North Dakota State University, Fargo, ND 58102 USA; 4 Department of Electrical Engineering, College of Engineering, Nanjing Agricultural University, Box 96, 40 Dianjiangtai Road, Pukou, Nanjing, China

**Keywords:** Remote-sensing information, Crop phenotype, Precision agriculture, Real-time and online, Variable-rate fertilization

## Abstract

**Background:**

Variable-rate fertilization is crucial in the implementation of precision agriculture and for ensuring reasonable and efficient fertilizer application and nutrient management that is tailored to local conditions. The overall goal of these technologies is to maximize grain output and minimize fertilizer input and, thus, achieve the optimal input–output production ratio. As the main form of variable-rate fertilization, real-time variable-rate control technology adjusts fertilizer application according to the growth status and nutrient information of crops and, as such, its effective application relies on the stable and accurate acquisition of crop phenotypic information.

**Results:**

Due to the relationship between crop phenotype and real-time fertilizer demand, phenotypic information has been increasingly applied in these contexts in recent years. Here, the establishment and characteristics of inversion models between crop phenotypic information and nutritional status are reviewed. The principles of real-time monitoring applications, the key technologies relating to crop phenotypic biological parameters, and the existing challenges for real-time variable-rate fertilization technology are also evaluated. Future research directions are then discussed in the specific context of the need for sustainable development of modern agriculture in China.

**Conclusion:**

This paper provides a theoretical reference for the construction of scientific management technology systems aimed at reducing fertilizer application and maximizing output, and for the development of relevant technologies in the specific context of China.

## Background

Scientific, rational, and effective fertilizer application plays an extremely important role in maximizing crop-yield potential, promoting grain production, and ensuring food security. It is, therefore, inevitably in demand for realizing the sustainable development of modern agriculture [1, 2]. Variable-rate fertilization, as a pivotal technology and the foundation of precision agriculture, has presented a new and effective way to meet the ‘green planting’ demands of modern agricultural with on-demand input and balanced fertilization [3]. Changing the large-scale and extensive fertilization management approach of traditional agriculture, variable-rate technology is supported by “3S” technology (i.e., remote sensing [RS], Geographical Information Systems [GIS], and the Global Positioning System [GPS]) to acquire real-time crop growth and nutrient gain–loss information as the basis for on-demand and variable-rate fertilizer inputs. In China, this modern approach is helping to balance soil fertility, overcome regional differences in biochemical parameters, improve fertilizer utilization rates and crop productivity, obtain higher crop yields and economic gains [4, 5], and promote the sustainable mechanization and automation of modern agricultural production.

**Fig. 1:**
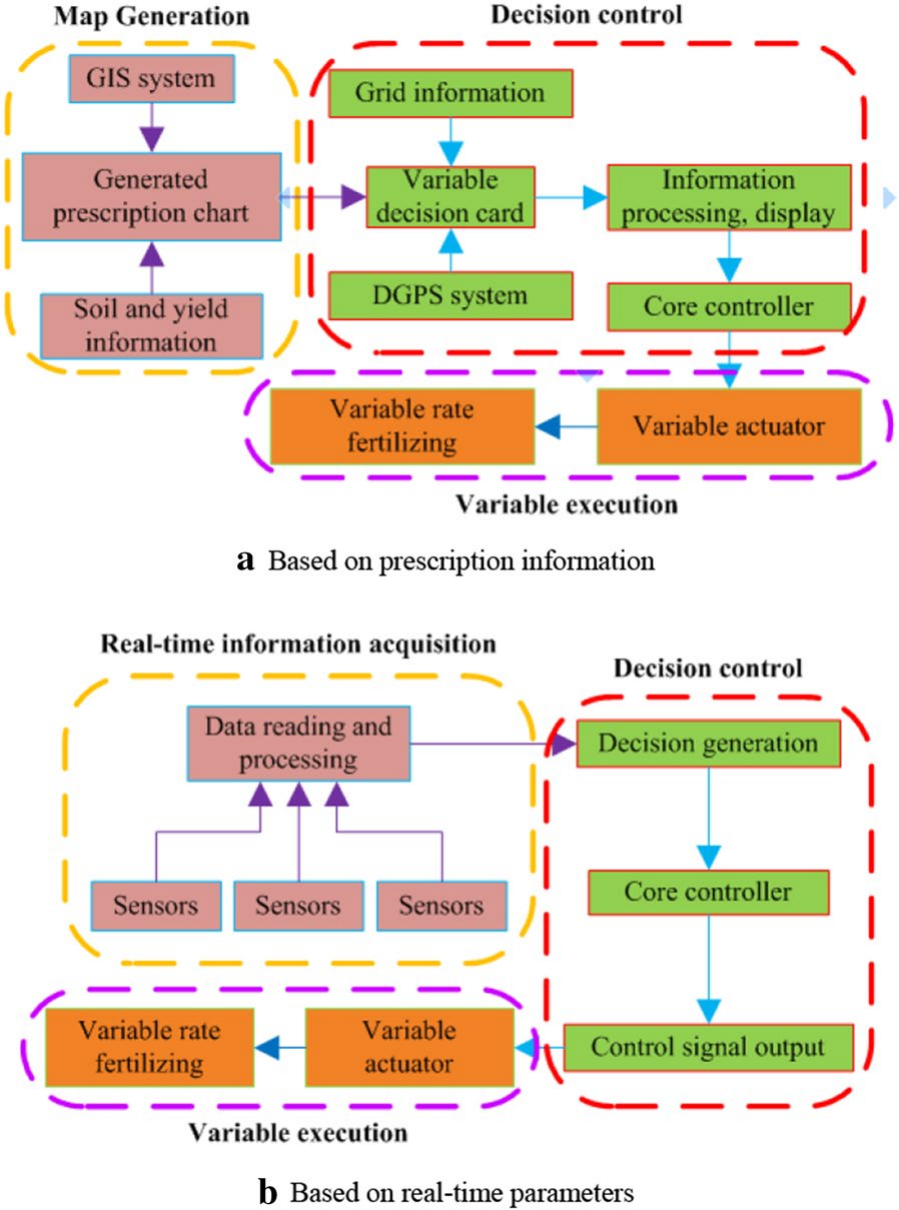
Technical flowchart of the variable-rate fertilization process

Since the concept of “precision agriculture” was advocated and implemented by American agricultural producers in the 1980s, variable-rate fertilization technology has developed rapidly in the field of intelligent agricultural equipment innovation in developed countries [6, 7]. The core control technology is largely based on two forms, i.e., prescriptive information and real-time parameters (Fig. 1) [8, 9]. The variable-rate fertilization control method based on prescriptive information (because of the limitations of the complicated process, difficult operation and high economic costs) cannot apply in scientific and reasonable variable fertilization according to the real-time nutrient demands during crop growth cycles [10]. However, variable-rate fertilization control technology (VRT) based on real-time parameters avoids the above-mentioned intricacy process, can be used to address problems associated with uneven growth and unbalanced soil nutrients. By capitalizing on modern, real-time sensor equipment, soil nutrient parameters and crop growth information can now be monitored online to generate target fertilizer applications in real-time [11], and scientifically guide the operation equipment to achieve on-demand, variable-rate fertilization.

The critical factor in the success of this modern approach (VRT) in the acquisition of stable, representative, and accurate field information including crop phenotypic information [12]. Studies have shown that there is a general relationship between real-time fertilizer requirements of crops and the phenotypic nutritional status of their corresponding growth stages, and that the real-time growth is closely related to the biological elements characteristics of leaves [13]. Crucially, the biological characteristics of crops show significant correlations with canopy spectral reflectance information [14]. Thus, the reflectivity and spectral parameters of different wavelengths have different quantitative relationships with leaf nutrient elements (e.g., nitrogen, phosphorus, and potassium content). The chlorophyll index (SPAD), leaf area index (LAI), ratio vegetation index (RVI), normalized difference vegetation index (NDVI), and other indexes can predict crop phenotypic parameters fairly well [15–17] including reflecting real-time growth information, that can be used to guide scientific and reasonable topdressing operations. Therefore, the appropriate fertilizer applications required for crops can be estimated by the effective models of statistical analysis based on phenotypic, biological parameters [18, 19].

In recent years, with the continuous development of computer science and remote sensing (RS) technologies, new ideas and methods for real-time, non-destructive nutrient detection during crop growth have been developed [20]. Thus, portable high-throughput phenotypic monitoring platforms combining agricultural RS technology and crop growth bio-indexes have emerged [21]. By carrying different types of real-time sensors, it is now possible to quickly, accurately, and non-destructively obtain multi-source RS data from which crop information can be quickly derived [22, 23]. At present, many well-developed products are widely applied in real-time variable-rate spreading operations [24, 25], including MSR-16 radiometers produced by American Cropscan Company, Crop Circle ACS-470 crop canopy sensors from the Holland Scientific Company in the United States, and GreenSeeker spectral sensors jointly developed by the American Trimble Company and Oklahoma State University. Considerable exploratory research has also been carried out in China [26].

In this context, this review combines current international research progress in variable-rate fertilization based on crop phenotypic information [27–29] with research on this technology being undertaken by our research group in China [30–32]. Specifically, the characteristics of inversion models between crop phenotypic information and nutritional status are evaluated, and real-time monitoring principles and key technologies associated with crop phenotypic parameters are identified. We also evaluate and discuss trends in the application and development of these technologies. Current limitations in the development and application of real-time variable-rate fertilization based on crop phenotypic information are highlighted, and feasible suggestions are made to provide a theoretical basis for future development in the specific context of China.

### Transmission mechanism of crop biological information

Remote sensing as an advanced, practical detection technology has the unique advantages of being rapid, non-destructive, multi-platform, multi-resolution (time and space), and providing access to ground and terrain information across large areas [33]. Since its inception in the early twentieth century, there has been rapid and on-going development of RS technology and it has been widely used in many fields, among which agricultural production is one of the most important areas. Agricultural RS technology plays an important role in agricultural resource surveys, biological yield estimations, and agricultural disaster prediction and assessment. Especially in recent years, the advantages of agricultural RS technology (i.e., offering the means to acquire timely, objective, and accurate information on crop area, growth, and yield) have been particularly important in China in view of the regional complexity and dispersion of crop distributions [34].

At present, agricultural RS technology has been widely used to measure land cover and crop biomass, and in particular, for the extraction of key crop biological and physicochemical parameters. The theoretical basis of RS is the targeted detection of spectral characteristics differences of electromagnetic waves based on crop phenotypes [35, 36]. Research has found that when exposed to electromagnetic waves, the spectral reflectance, absorption, transmission, and emission characteristics responding in different wavebands differ between crops [37]. Especially in the visible-near-infrared spectrum bands, reflectivity is mainly affected by phenotypic pigments, cell structure, water content as well as other biological parameters. There are strong absorption characteristics in the visible red light wavebands and strong reflection characteristics in the near-infrared wave bands, which are often used for monitoring crop growth, production yield and quality, and pests and diseases [38]. Generally, spectral reflectivity is the ratio of the reflected energy of an object to the incident energy at a particular wavelength interval. When the solar spectrum with a wavelength of *λ* is projected into the crop canopy in parallel, Lambertian reflectance occurs [39], thus the reflection model of crop phenotype conforms to Lambert’s cosine law, as follows:

1 $$ \frac{{d\phi_{\lambda } }}{dA \cdot d\varOmega } = L_{\lambda } \cdot cos\theta , $$

where *φ*_*λ*_ is the radiant flux (i.e., the radiant energy passing through a section per unit time); *L*_*λ*_ is the radiation luminance of the crop canopy; *A* is the projection area of crop canopy; *Ω* is the solid angle; and *θ* is the reflection angle.

Using Eq. 1, the total radiated power per unit area of crop canopy to the hemispherical space can be calculated. That is, the radiant exitance (*M*_*e*_) and the relationship between reflection radiation luminance (*L*_*λ*_) of the crop canopy can be derived as follows:

2 $$ \begin{aligned} M_{e} &= \int {\int {\frac{{d\phi_{\lambda } }}{dA}} } = \int {\int {L_{\lambda } \cdot cos\theta d\varOmega } } = \int_{0}^{2\pi } {\int_{0}^{{\frac{\pi }{2}}} {L_{\lambda } \cdot cos\theta \cdot sin\theta d\theta d\varOmega } } \\        &= \int_{0}^{2\pi } {\int_{0}^{{\frac{\pi }{2}}} {L_{\lambda } \cdot \frac{sin2\theta }{4} \cdot d\left( {2\theta } \right)d\varOmega } } = \pi L_{\lambda }  \\ \end{aligned} $$

If the radiant flux of the solar spectrum with a wavelength of *λ* parallel to the unit area of crop canopy is *E*_*λ*_, then the spectral reflectivity (*ρ*_*λ*_) of the crop canopy with respect to the solar spectrum is derived as follows:

3 $$ \rho_{\lambda } { = }\frac{{M_{e} }}{{E_{\lambda } }} = \frac{{\pi L_{\lambda } }}{{E_{\lambda } }}. $$

Real-time variable-rate fertilization technology applies these radiation transmission mechanisms to obtain biological information about crop phenotype, nutrient use, and production yield quality. This approach depends on accurate determination models for different nutrient and biological elements inversed by RS technology, such as the nitrogen/chlorophyll concentration, nitrogen/chlorophyll accumulation, leaf area index, biomass, and canopy density [40]. Furthermore, along with expert decision systems built into variable-rate fertilization equipment, scientific and rational nutrient regulation and fertilizer management can be enabled. This effectively solves the problems associated with regional differences of biochemical parameters of field crops, improves fertilizer utilization efficiency and crop production capacity, and can support precision agriculture modernization and sustainable development.

### Inversion between crop phenotypic information and nutritional status

With the continuous development of agricultural RS technology, it has increasingly been applied in crop phenotypic monitoring and the diagnosis of plant nutritional status in large-scale agricultural production. This includes growth detection and yield prediction of wheat, corn, rice, cotton, and other staple crops [41, 42]. The inversion of crop growth information (i.e., plant nutritional status) can be acquired by monitoring crop canopy biological characteristics [43, 44]. This relies on the internal relationships between crop features (e.g., canopy geometry, leaf biochemical composition, and internal tissue components) and canopy spectral reflectance characteristics [45, 46]. Data are obtained through spectral data mining (e.g., statistical discriminant, correlation analysis, and regression modeling) to establish corresponding algorithm and metrics, such as the crop nitrogen nutrition index (NNI), ratio vegetation index (RVI), and leaf nitrogen accumulation (LNA) [47].

Currently, the commonly used inversion methods are mainly divided into two categories [34, 48]. First, the empirical statistical method is based on the relationships between crop phenotype biological parameters and spectral reflectivity or other spectral characteristics in sensitivity bands. This is a relatively simple approach; however, established empirical relationships rely excessively on specific conditions, such as the measurement instrument, measurement time, crop variety, and environmental environment, meaning that applicability can be limited [49]. Second, the radiation transmission model inversion method is based on RS monitoring process, commonly used numerical optimization algorithms, the partial least-squares method, principal component analysis (PCA), support vector machine training sets, and the artificial neural networks. These techniques are applied to simplify the derivation process and improve inversion efficiency, which has a stronger universality [50, 51]. The application of these methods in research on high-throughput biological monitoring and nutrient status discrimination of crop phenotypes has included multiple scales, from crop populations to plant tissues and organs, and has yielded rich and diverse results [52].

Crop canopy cover is a phenotypic parameter that quantitatively describes the dynamic differences in growth and development between genotypes. It is an important indicator to characterize and evaluate crop nutritional status (such as nitrogen status, early vitality, senescence state, and surface biomass) and has important applications in monitoring crop growth [53]. Currently, there are many indexes that are closely related to crop canopy cover, of which the NDVI and the LAI are the most well-developed [54, 55]. The NDVI can be monitored in real-time and has been applied as an objective means of (indirectly) assessing variations in the characteristics of wheat under stress and seasonal nitrogen demand [56]. It can also be used to estimate crop nitrogen content, surface nitrogen distribution, and crop nitrogen utilization efficiency. The close correlation between NDVI and crop physiological characteristics can also be used to explain the diversity impact factors on crop production, such as changes in moisture and nitrogen regimes during different growth stages [57, 58]. Li et al. [59] discussed the relationship between the NDVI and crop LAI using Thematic Mapper (TM) imagery and directly used the inversed LAI as the growth grading standard to monitor the growth of winter wheat in Xinghua City, Jiangsu Province, China. Their study also assessed indexes of crop nitrogen content and nitrogen demand application to guide scientific and rational variable application of nitrogen fertilizer on demand, this improves the level of fertilizer utilization efficiency and produces significant economic benefits.

Nitrogen nutritional status and nitrogen demand application are central in the monitoring and diagnosing of crop growth (and the important mainstay for making management decisions of field production), and provide the necessary technical basis for achieving the modernization and sustainable development of precision agriculture [60, 61]. Through experimental studies, Shibayama et al. [62] showed that the nitrogen content per unit area of crop leaves had strong regression relationships with reflectivity in spectral bands R400, R620, R760, and R880, which were not affected by crop type or variety. Liu et al. [63] used the characteristics of the reflection peak at 1690 nm to invert the carbon–nitrogen ratio of wheat canopies at each growth stage. This enabled a segmented RS inversion model of leaf and stem-sheath to be established for the rise and milk stages, and the accuracy of estimating the nitrogen content in fresh leaves using spectral analysis method was improved to > 85%. Cho et al. [64] proposed a linear extrapolation method to calculate red edge position information about crop canopy reflectance spectra, and established models for predicting the nitrogen concentrations of wheat plants, maize leaves, and mixed pasture plants. Chen et al. [65] showed that the dual-peak canopy nitrogen index (DCNI) eliminates the interference of leaf area changes with respect to the diagnosis of nitrogen concentrations and, thus, enables the accurate detection of plant nitrogen concentrations. Furthermore, Chen et al. [66] were able to derive the nitrogen nutrition index (NNI) of winter wheat and maize using RS techniques, which could well determine the nitrogen nutrition status.

With the advancement of research on crop phenotypes and nitrogen nutrition, increasing attention has been paid to the heterogeneity of vertical distribution of nitrogen in crop canopies, nitrogen content at different height of the crop canopy plays an important implications for scientific guidance of variable-rate fertilization and field management [67, 68]. For example, Wang et al. [69] used a partial least-squares (PLS) algorithm to invert the nitrogen concentration of leaves at different canopy levels based on the vertically observed canopy spectra of winter wheat. Wang [70] also estimated the nitrogen density of different leaf layers using the difference vegetation index (DVI) of winter wheat combined with the interception of luminous energy at each canopy level. Zhao et al. [71] achieved higher accuracy estimates by constructing an inversion index of chlorophyll concentrations in the upper, middle, and lower leaf layers of winter wheat based on multi-angle canopy reflectance spectra. Based on field experiments and RS, Ma et al. [72] further analyzed the correlation between nitrogen concentrations and the spectral and fluorescence characteristics of leaves at different vertical heights, which provided a new idea for exploring the RS method to determine the vertical distribution of crop canopy nitrogen.

Using field-based equipment, observation towers, and hand-held sensors as carriers (according to the acquired phenotypic biological information and inversed nutrient evaluation models), rational on-demand nitrogen application and regulation management can be achieved. Such equipment includes radiometers (e.g., the MSR-16), nitrogen sensors (e.g., the Yara N-sensor), crop canopy sensors (e.g., the Crop Circle ACS-470), spectral sensors (e.g., GreenSeekers), and non-imaging spectrometers (e.g., the ASD Fieldspc FR2500) [73–75]. Specific examples include Kitchen et al. [76], who applied the green channel NDVI (GNDVI) obtained using the ‘Crop Circle’ device to monitor crop nitrogen deficiency, and then guided the variable-rate fertilization. Their results showed that the method could be used to reduce the nitrogen application and improve the crop yield. Peng et al. [77] also proposed a means of real-time nitrogen management (RTNM) based on measured crop SPAD values to rapidly determine nitrogen nutritional status. Scarf et al. [78] estimated the nitrogen nutritional status of maize in an unfertilized area based on the difference in corn greenness values between a saturated and control fertilization area, and used this as a basis for variable-rate fertilization management. Lukina et al. [79] used canopy NDVI to invert the nitrogen accumulation of winter wheat to estimate potential yield under variable-rate fertilization management. Their research was developed further by Chen et al. [80], who used field object spectral data to improve the universality of the nitrogen application optimization algorithm. Zhao et al. [81] also established a variety of variable-rate fertilization algorithms for winter wheat based on the optimized soil-adjusted vegetation index (OSAVI) and chlorophyll (SPAD) values. Their results showed that the economic and ecological benefits of variable-rate fertilization based on the spectral data are superior to other traditional fertilization algorithms based on the soil fertility.

### Real-time variable fertilization systems based on crop phenotypic information

**Fig. 2:**
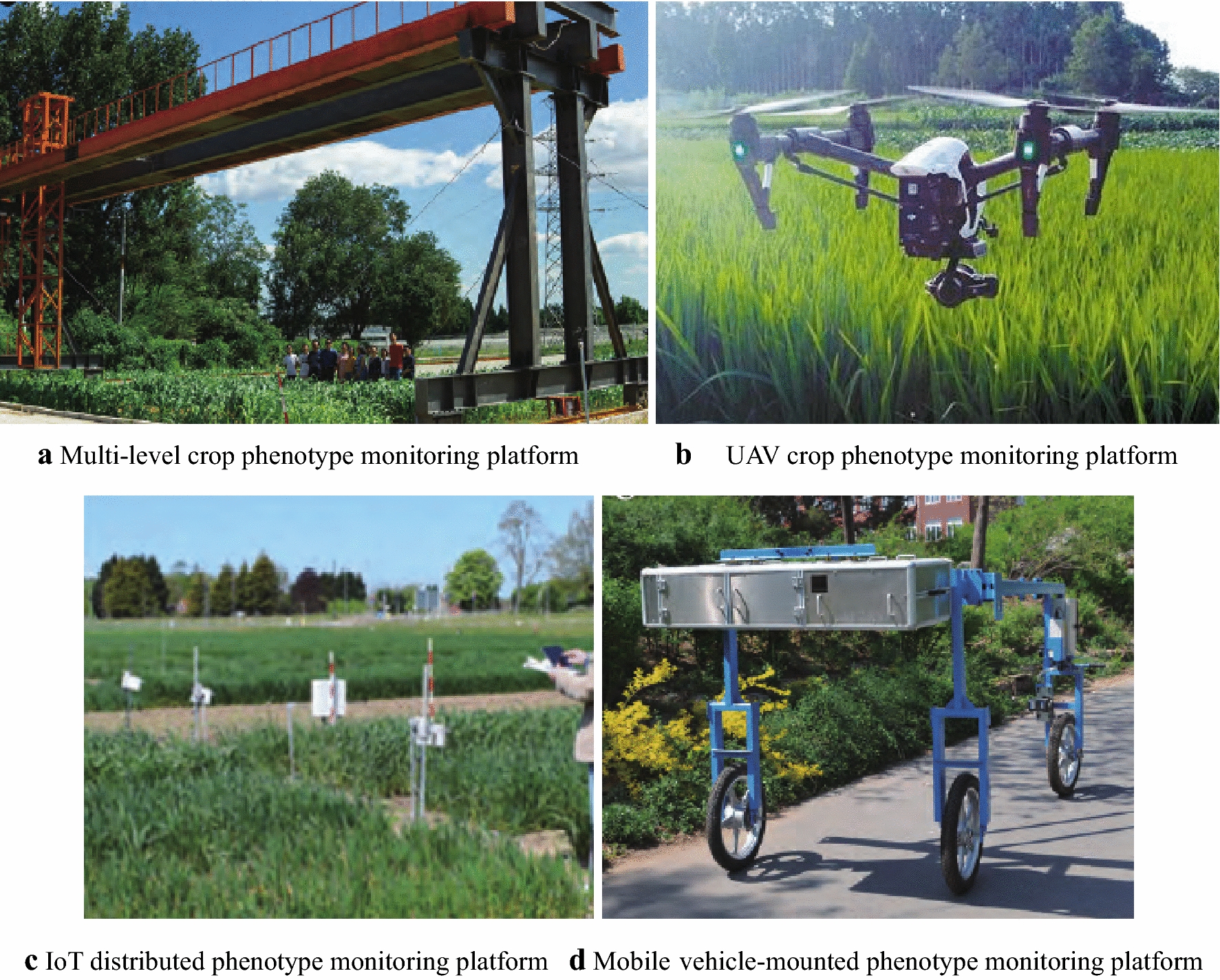
Example phenotype monitoring platforms for field crops

Based on the continuous development of computer science and RS technologies, and the agronomic requirements of different types of crops, detection platforms for the acquisition of biological information from crop canopies have been increasingly applied in real-time variable-rate fertilization [40]. According to canopy biological parameters obtained using real-time monitoring systems, expert decision-making systems are then used to determine the target fertilizer application online. Once determined, the core controller adjusts the actuator, which controls the actual application of fertilizer to the crop. Relative to traditional approaches to fertilizer application, such set-ups improve fertilizer utilization efficiency, increase crop yields and economic gains, and improve ecological standards. In accordance with the different application carriers, phenotypic monitoring platforms are mainly divided into two types: (1) aviation-based and (2) ground-based approaches (Fig. 2). Common aviation-based phenotypes monitoring technologies are divided into satellite-borne and airborne types, and ground-based technology can be divided into hand-held and vehicle-mounted types [82].

Multi-source satellite images acquired using the satellite-borne platforms can be used to extract crop phenotypic parameters; however, due to the limited time and spatial resolutions of data acquisition, it is difficult to apply over small-area test regions and for high-frequency dynamic monitoring [83]. In comparison, airborne phenotypic monitoring platforms (e.g., helicopter or unmanned aerial vehicle [UAV]) carrying multiple sensors simultaneously, can acquire multi-source image information for entire test sites in a relatively short period of time [84–86]. This approach has been applied to measure the canopy temperatures and lodging situations in thousands of field plots as well as canopy structure at various test sites [87, 88]. UAVs or drones are now used to carry multispectral cameras (such as the Mini-MCA6), hyperspectral cameras (such as the Cubert UHD185) and active non-imaging sensors (such as the RapidSCAN) that enables the derivation of the LAI, aboveground biomass, and nitrogen nutrient status for crops such as wheat, rice, cotton, and corn [89–91]. For example, in Japan, TM-based RS data has been used to monitor nitrogen, amylose, amylopectin, and other quality indicators in rice, which has been used to guide the application of nitrogen fertilizer [92]. This reduced the nitrogen content of rice grains in a larger experimental area from 7.7 to 7.3% and thereby improved crop quality economic gains. However, airborne platforms involve higher costs, require more technical maintenance effort, and the RS information obtained by individual sensors is often limited. Multi-sensor coordination and data fusion are, therefore, required. Moreover, canopy disturbance effects become problematic when acquiring high-resolution images at low altitudes [93–95]. Ground-based phenotypic platforms usually involve common agricultural equipment adapted with a series of phenotypic sensors and a GPS for positioning and navigation [96, 97], these platforms can simultaneously acquire multi-source information (such as multispectral, hyperspectral, and thermal infrared images). As different sensor groups can vary greatly at different test points and under variable environmental conditions, therefore, means of calibrating sensor groups are required to ensure reliability and comparability, which is the main technical challenge when using this vehicle-based phenotype platform [98].

At present, data acquisition technology providing phenotypic information for field crops (widely applied in real-time variable-rate fertilization for precision agriculture) is still dominated by ground-based and vehicle-mounted operations. Vehicle-mounted phenotypic and omics platforms are simple to operate and can obtain high-resolution and continuous spectral data from close-up observations. Sensors are mounted at different locations and heights of the platform depending on the crop type and growth period [99]. Related accessories include power systems, data acquisition terminals, GPS receivers, and encoders to ensure the running work of the vehicle. A high-performance terminal that can receive various sensor data is also needed. When equipped with a Beidou satellite, radar, GPS, gyroscope, and other sensors, vehicle-mounted platforms can achieve automatic navigation, generate distribution maps of crop phenotypic features, and coordinate with the optimized expert decision system to conduct reasonable field nutrient management [100, 101]. Currently, intelligent agricultural equipment for variable-rate fertilizer application systems are relatively well-developed, and a wide range of technical equipment has been commercialized and applied internationally and at a large-scale.

**Fig. 3:**
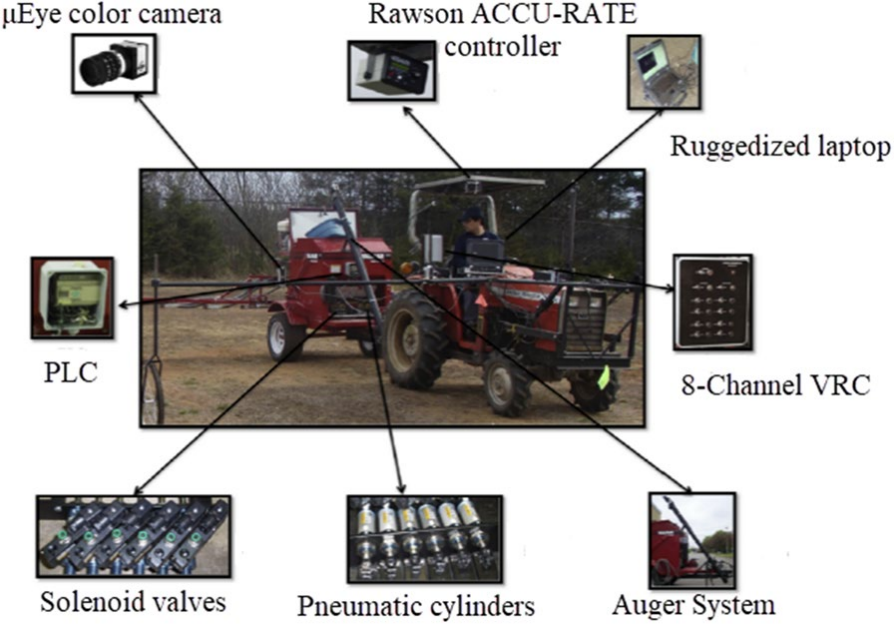
Variable-rate spreader for wild blueberry

**Fig. 4:**
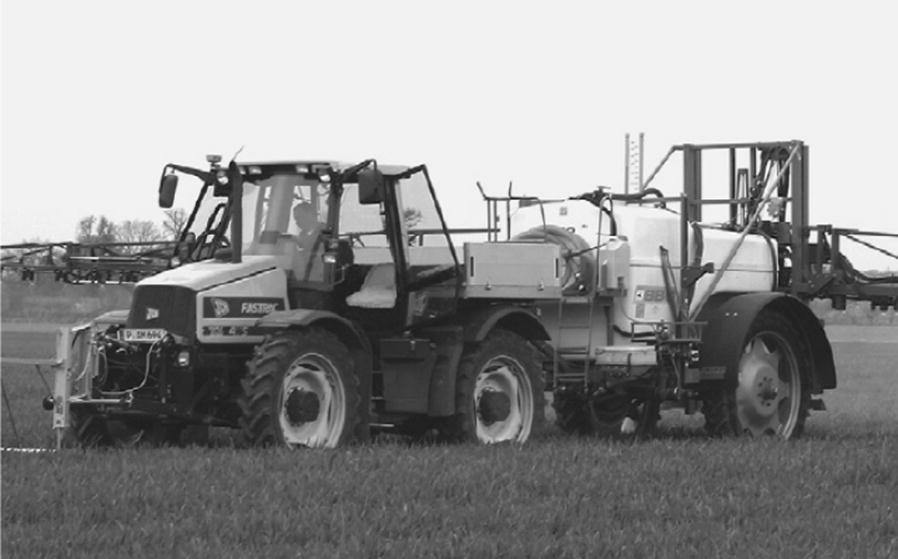
Variable-rate fungicide sprayer

Zaman and colleagues [102] used a color camera (with wireless Bluetooth communication) to obtain biological data for wild blueberry canopies in Canada, which they used to respond to the corresponding control procedures to adjust fertilizer inputs. That achieve efficient, reliable, and accurate variable-rate fertilizer with corresponding economic and ecological benefits (Fig. 3). Dammer et al. [103] developed a fungicide variable sprayer in Germany based on the LAI of cereal crops, as shown in Fig. 4. In their system, crop biomass density is measured in real-time by a portable spectral sensor (CROP-Meter) fixed at the front of a tractor. GPS information is then combined to generate a control signal for sterilization doses administered via an automated, adjustable hydraulic valve to achieve variable-rate spraying.

**Fig. 5:**
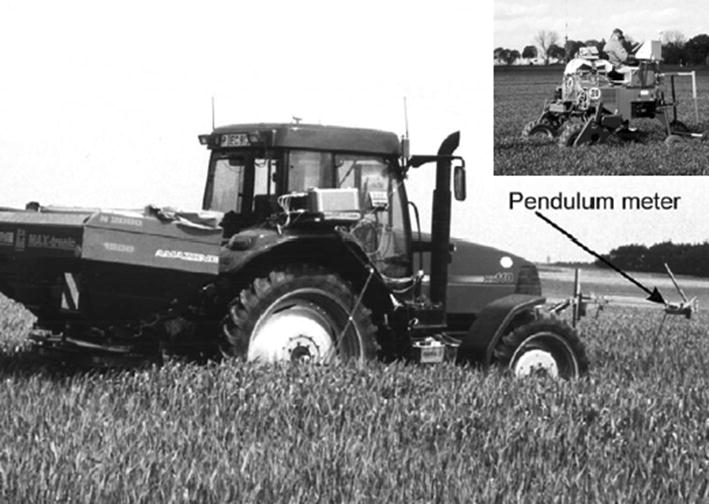
Variable nitrogen fertilizer of winter wheat

**Fig. 6:**
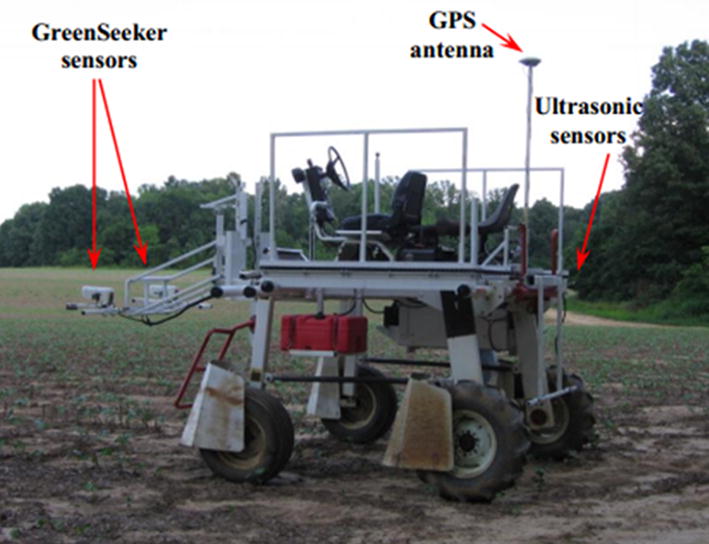
Variable-rate fertilizer applicator for cotton

Ehlert et al. [104] developed a variable-rate nitrogen fertilizer spreader for winter wheat based on a crop density detector with a mechanical pendulum (Fig. 5). In their system, an onboard computer continuously adjusts fertilizer application based on crop density parameters measured at a fixed point. Multiple field trials using calcium ammonium nitrate fertilizer (nitrogen fertilizer) under different seasons showed that this system could reduce fertilizer application by 10‒12% and improve crop yield and quality. To improve the utilization rate of nitrogen fertilizer during the cotton production process in Tennessee, USA, and to optimize the impact of soil reflection using the GreenSeeker sensor, Mariso [105] designed an optimization algorithm and an ultrasonic device to control spraying distance form cotton canopy, as shown in Fig. 6.

**Fig. 7:**
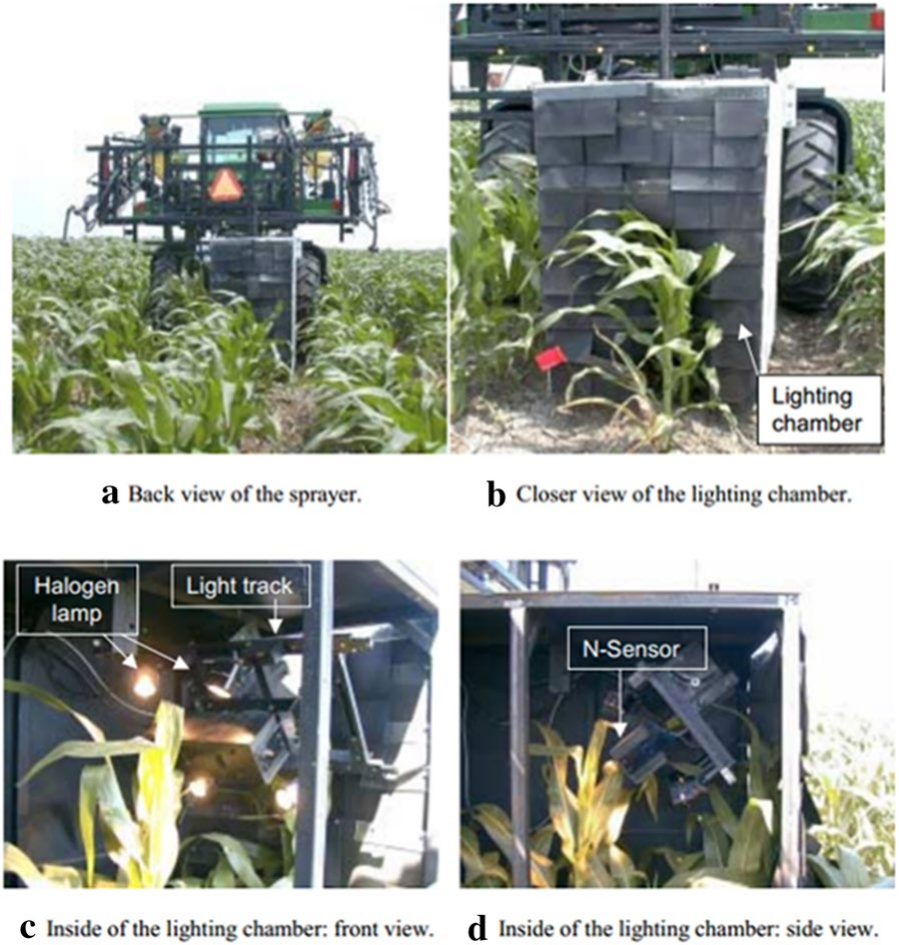
Variable-rate fertilizer applicator for corn

**Fig. 8:**
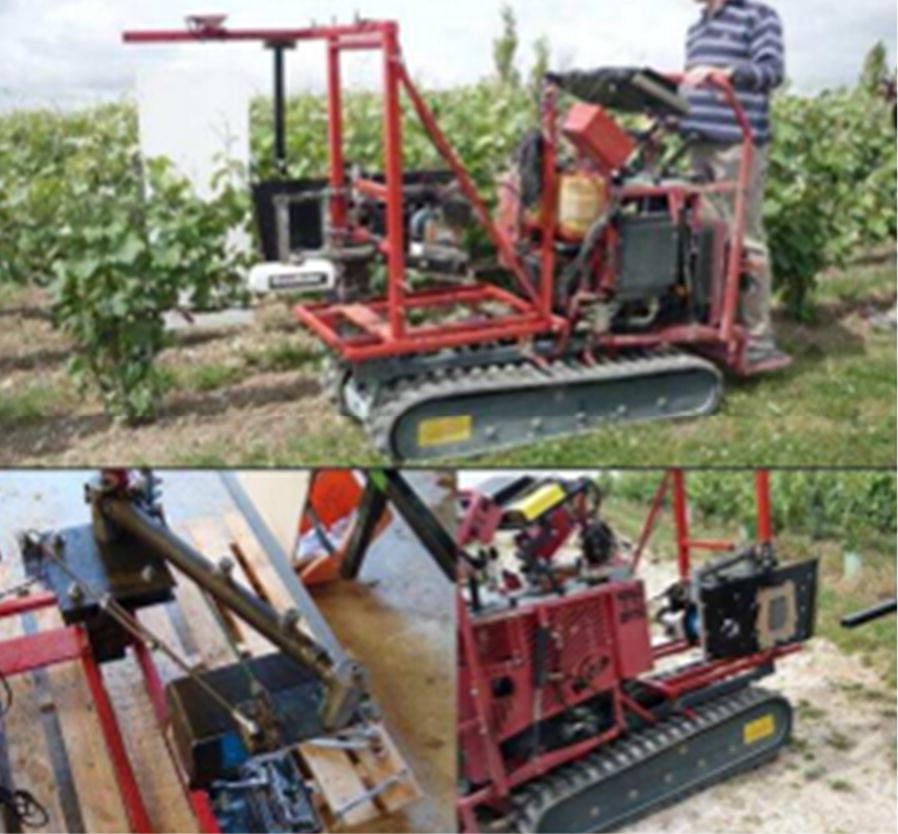
Variable fertilizer applicator for grape vines

A variable-rate fertilization machine based on spectral detection technology was developed for maize by Lee and Searcy (Fig. 7) [106]. In their set-up, a spectrum monitoring system including a halogen lamp and an N sensor was installed behind a tractor to monitor the nitrogen nutrition status of corn in real-time. GreenSeeker sensors and Multiplex sensors have also been used to monitor the nutritional status of grapes in real-time and to guide field fertilizer management [107] (Fig. 8). In this case, the system was able to obtain various field indices including the chlorophyll fluorescence index (SFR), nitrogen balance index (NBI), and flavonoid index (FLAV).

In China, research on precision agricultural production and variable-rate fertilization technology has been relatively slow to progress and remains in its infancy. Recent developments have mainly resulted from the introduction of foreign advanced agricultural machinery and their supporting control systems, although there remains a lack of systematic theoretical research and related experiments in this field [108]. In recent years, with the continuous development and improvement of relevant national policies including the ‘Application Reduction and Efficiency Increase’ policy, independent and innovative research in intelligent agricultural machinery and control systems has begun to progress rapidly with groping and exploring of precision agricultural in succession.

**Fig. 9:**
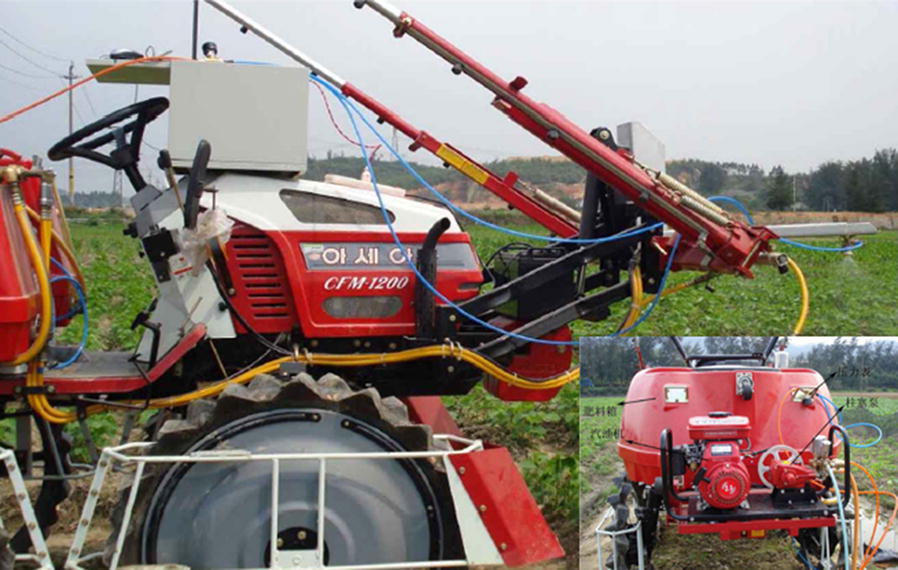
Variable-rate fertilizer machinery for soybean crops

**Fig. 10:**
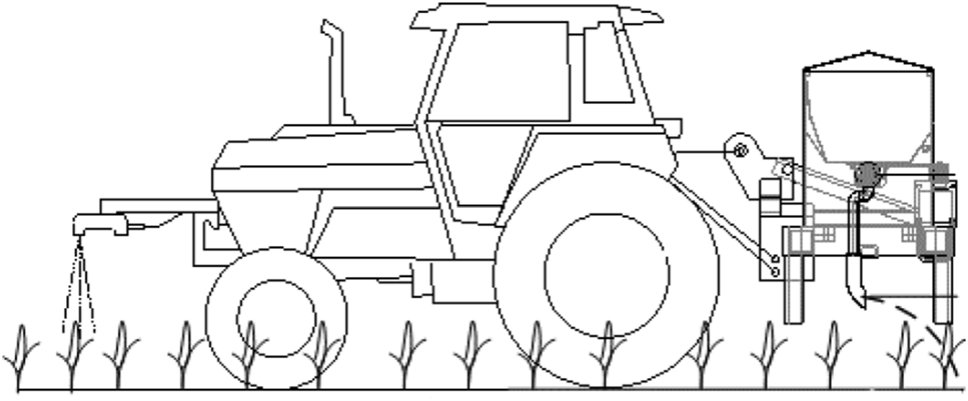
Schematic of a variable-rate fertilizer machine based on crop growth

Examples include Xu et al. [109] who used an MSR16R multispectral instrument to obtain canopy spectral reflectance information for soybean crops. They established a nutrition diagnosis model to estimate the required levels of nitrogen for growth and an expert decision-making system for stable, variable-rate fertilization based on fuzzy control theory (Fig. 9). Zhang et al. [110] developed an online measurement variable-rate fertilizer applicator based on crop growth (Fig. 10). They used non-destructive, photoelectric inspection technology to obtain real-time NDVI data and a core processor controlled different fertilizer applications based on a fuzzy control algorithm. Based on the correlation between crop canopy spectral reflectance and growth, their system achieved the real-time online ‘living diagnosis’ of community growth trends and the LAI across the whole crop growth period. Their system was able to guide reduced fertilizer application in areas of stronger crop growth, and more fertilization in areas with poor growth, thus enabling precise variable-rate fertilization according to crop growth.

**Fig. 11:**
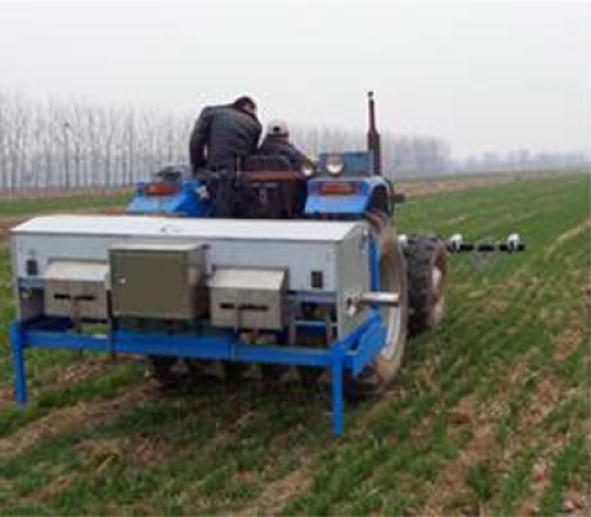
Variable fertilizer based on canopy spectral reflectance

**Fig. 12:**
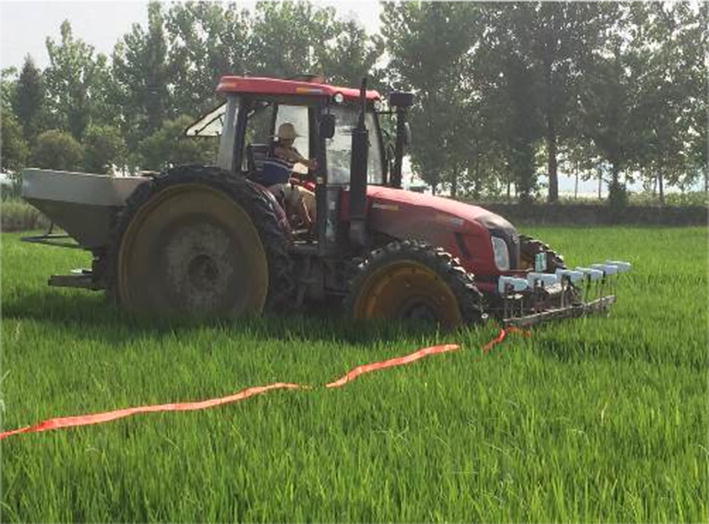
Variable spreader for rice

Wang and colleagues [27] evaluated chemical (nitrogen) fertilizer demand based on NDVI values of winter wheat using real-time monitoring and implemented an intelligent control program for variable-rate fertilization (Fig. 11). At the same time, their decision-making tool provided real-time targeted top-dressing applications, realized the online adjustment of the fertilization amount [27, 28]. Furthermore, Shi et al. [29] developed a centrifugal and high-efficiency variable-rate fertilizer spreader based on real-time growth information for rice (Fig. 12). In this case, a GreenSeeker spectral detection system obtained real-time DNVI values of rice canopies and was coupled with the previously established expert decision-making system to generate target fertilizer requirement application, then real-time spreading application was adjusted online [30–32] to realize accurate variable-rate fertilizer spreading for rice.

It can be seen that real-time variable-rate fertilization technology based on the vehicle-mounted phenotype omics platform has great application potential in the research of crop phenotype bioinformation monitoring and variable-rate fertilization technology research of precision agricultural. Moreover, of course, other vehicles that can be mounted with sensors and high-throughput phenotypic acquisition tools include airships, parachutes, hydrogen balloons, and ground observation towers, but these are limited by their inflexibility (such as line spacing, plant spacing, and crop height limitations) and cost, which makes them difficult to apply across different crops and at different growth stages. Furthermore, the image resolution, safety performance, and load capacity of these other approaches all need to be improved further [16, 40].

## Results

In recent years, research into phenotypic information monitoring, precision agriculture, and variable-rate fertilization technologies has progressed in China. However, real-time variable-rate fertilizer systems and its application based on crop phenotype bioinformation are still at the experimental stage being currently limited from the following key challenges:

1. The study object, monitoring area and scope are relatively limited. At present, research on variable-rate fertilization technology based on crop phenotypic biological information mainly focuses on specific field conditions and crop varieties. However, given that China has a relatively developed agricultural economy and a substantial cropping area, research into the application of precision agriculture that accounts for regional and local features is lacking, especially for multi-species crops in agricultural underdeveloped areas. For example, most of the existing research targets wheat, corn, rice, barley, and other cereal crops while very few studies have been reported for beans, potatoes, and other important commercial crops.
2. Obtained biological parameters of crop phenotypes are single. Each imaging technique has its own limitations that are affected by temperature, illumination, and other complex field conditions. Some RS techniques are limited by their measurement accuracy, and spatial and spectral resolution are yielding large measurement errors, and they cannot be widely applied. It is, therefore, necessary to explore the fusion of different phenotypic data sources, at different stages of data processing, to overcome or reduce detection error associated with single-source approaches. This is essential to improve the accuracy of crop phenotypic parameters and the stability of variable-rate fertilization equipment.
3. Rapid processing of the enormous RS data faces huge challenges. Hyperspectral imagers and Lidar sensors rapidly acquire large amounts of high-temporal resolution information. The processing of these datasets is, therefore, limited by computer hardware performance and processing power. It remains difficult, for example, to achieve rapid online processing of ‘big data’ in real-time, and the mixed spectra of crops at different growth stages along with variable soil compositions adds significant complexity. Indeed, the mixed spectra of field components are difficult to decompose, resulting in lower accuracy targeting of fertilizer requirements for inversion calculations.
4. Inversion models of fertilizer requirement is lacking in common methods. For practical application, when parsing phenotypic information for different crops and under different growth stages, modeled needs to be performed separately, which ignores the synergistic influence of multiple physiological parameters. The applicability of models is also limited by environmental conditions and variability and uncertainties, such as the driving variables or parameters of RS inversion models, and uncertainty associated with error propagation. This leads to crop growth models and nutrient recommendation systems that cannot be closely matched with variable-rate fertilization machinery and, hence, the cycle of expert-decision analysis in the system slows down.
5. Currently, intelligent control systems have little supporting research and limited development capabilities. Many sensors remain expensive, vehicle-mounted hyperspectral sensors, laser radar sensors, and chlorophyll fluorescence sensors must all currently be imported to China, resulting in high costs. This also means that the core technology of these sensors cannot be easily adapted, optimized, and upgraded, limiting the applicability under specific operational demands of the Chinese agricultural sector. Detection sensors for crop phenotypic monitoring with independent intellectual property rights are also lacking, which restricts the extensive promotion and application of variable-rate fertilization equipment based on crop phenotypic information in modern planting areas in China.

## Discussion

Whilst still at the exploration stage, as an emerging trend in precision agriculture, real-time, variable-rate fertilization technology based on crop phenotypic information will continue to develop in China into the future. Further research and promotion are required, especially for high-throughput phenotypic data acquisition and the integration and application of real-time variable systems. Further technical development and research collaborations are, therefore, needed. Building on the advanced experience of other countries and based on the specific machinery and agronomic requirements of Chinese agriculture, matching variable-rate fertilizer equipment with independent innovation should be targeted. This will reduce production costs and demonstrate and applicability in modern agricultural areas, thereby fostering the effective and efficient utilization of fertilizer resources to achieve healthy and sustainable development. In the future, we believe that real-time variable-rate fertilization techniques based on crop phenotypic information will continue to receive attention under the key themes outlined in the following sections.

### Continuously expanding the breadth and depth of crop phenotypic data acquisition, strive for the extraction of high-latitude parameters, and multi-scale analysis

The acquisition of crop phenotypic information based on RS technology has significant advantages and opportunities in the application of real-time variable-rate fertilization. Its flexibility, timeliness, and high efficiency are key characteristics necessary for crop phenotype information extraction and real-time variable-rate fertilization. However, current research on data acquisition using vehicle-mounted sensors mainly focuses on specific varieties of common grain crops while few have examined important commercial crops like beans and potatoes. The majority of existing studies are devoted to extracting or parsing single phenotypic parameters and lack the requirements of effective phenotypic identification and fertilizer requirement analysis in complex farmlands. In addition, the accurate description of crop nutrient status during growth depends on the acquisition of multi-dimensional data and its analysis at different scales. Therefore, it is necessary to continuously expand the breadth and depth of studies on parsing crop phenotypes using RS and devote effort to the extraction of high-latitude environmental parameters and multi-scale analysis. This will establish a theoretical foundation and technical reference for promoting the application of crop phenotypic monitoring using near-earth RS technology as applied to real-time variable-rate fertilization for precision agriculture.

### Deeply interrogate and integrate multi-source RS data to construct analytical models of high-throughput phenotypic information

A large number of narrow-band and continuous crop hyperspectral images obtained by RS technology can more comprehensively represent the unique spectral characteristics of crops and accurately derive their biophysical and biochemical states. Furthermore, hyperspectral data contains abundant information and requires a significant amount of data processing. However, at present, such datasets do not play an important role in growth state monitoring. It is, therefore, necessary to strengthen research in the use of hyperspectral RS for the analysis of crop phenotypic information. This includes the acquisition of data for crop three-dimensional size, biomass, and vegetation index as well as other indicators. Moreover, the majority of existing analysis models are constructed based on a single method, having limited wider applicability and poor inter-annual prediction stability. To increase the prediction accuracy of crop phenotypic information and improve nutrient inversion models, it is necessary to combine multi-source RS information (such as color, depth, and spectral data) with environmental and crop physiology knowledge, and apply machine learning and deep learning algorithms at the pre- and post-processing stages. Such an approach based on a variety of methods will help secure versatile, stable, and high-precision analysis models for high-throughput phenotypic information and recommendation models for fertilizer requirements application that can be subjected to systematic testing and verification.

### Persistently accelerate the development of high-resolution, low-cost sensors with independent innovation tailored to China’s national conditions

High costs and low spatial and spectral resolution data remain critical bottlenecks for the promotion and application of RS technology in crop phenotypic information detection. At present, most sensors, hyperspectral instruments, thermal imaging cameras, and other equipment carried on vehicle-mounted RS platforms in China are imported from Europe or the USA. The price of these products is inevitably high and their applicability for crop varieties grown across China’s agricultural regions is limited. Furthermore, the key core technologies cannot be optimized and upgraded for Chinese crops, which seriously restricts wider application. Therefore, in the course of future development, comprehensive strategy based on the actual planting, growth, and management characteristics of different regions in China is required. This must be combined with new fertilizer application methods tailored to the specific cropping requirements and fertilizer use of different regions. Developing the detection sensors with independent innovation ability for crop phenotypic in line with Chinese characteristics, and effectively promote the application of RS technology in phenotypic information monitoring for crops. This will ensure the necessary infrastructure is in place for successful real-time variable-rate fertilization systems that will facilitate the uptake of precision agriculture and green, healthy, and sustainable development.

### Effectively promote multidisciplinary and multi-field data-sharing platforms for crop phenotypes and fertilizing decisions

Research of crop phenotypes and precise, variable-rate fertilization has relevance to the fields of plant science, information technology, biotechnology, agricultural technology, and engineering technology, is a composite science and technology with multidisciplinary cross and multi-field integration. Multi-system, interdisciplinary authoritative expert knowledge and support decision-making system are therefore required. Based on the nutrient demands and balanced principle of different crop types and growth phases, combined with advanced technologies including network transmission, information perception, and data processing to complete the high-precision, comprehensive, and efficient coverage, detection and collection of soil and crop nutrient information, as well as the digital nutrition decision management systems. In addition, effective systems must account for the effective nutrient requirements of crops, plant growth, water conditions, visible symptomatic diagnosis, target yields, and fertilizer methods, therefore, it is urgent to establish a set of database-sharing platforms for phenotype-based fertilization decisions with multi-disciplinary cross and multi-field joint, effectively promotes the deep cooperation between different fields of research, provides a much more efficient route to bringing new transformative technologies into practice, and can jointly promote the development of precision agriculture in China.

### Profoundly intensify efforts to cultivate technical and practical talents for phenotypes detection and precision agriculture (real-time variable-rate fertilization)

The significant amounts of digital, spectral, and thermal imaging data that can be obtained by RS platforms require a series of advanced processing stages, such as geometric calibration, radiation correction, and data modeling. This requires practitioners to have the appropriate scientific and cultural information available to them and the technical experience to use the relevant digital and mechanical equipment. However, at present, end-users lack data-processing skills and this can prove a challenge when adapting current practices. Therefore, it is necessary to strengthen expertise in agricultural science and technology, to provide adequate financial support for agricultural research, and intensify efforts to cultivate high-quality applied experts in precision agriculture. Training, especially young and middle-aged farmers, is an important tool in promoting crop phenotypic research, the healthy development of precision agriculture, and for meeting the new challenges that the rest of the twenty-first century will present. At the same time, it is necessary to speed up the development and demonstration of full-process technical solutions that are easy to operate, and integrate different functional software that enables users to independently monitor crop growth, predict yields, and derive fertilization recommendations, promote real-time variable-rate fertilization technology based on crop phenotypic biological information nationwide in China.

## Conclusions

Since the concept of precision agriculture was advocated and implemented by American agricultural producers in the 1980s, variable-rate fertilization technology has developed rapidly in the field of intelligent agricultural equipment innovation. More recently, crop phenotypic information acquired using RS technologies has made a significant contribution to understanding crop growth, yields, and quality alongside environmental variables including natural disasters. This approach offers technical superiority in supporting the management of fertilizer applications, water use, and crop damage, and has gradually obtained industry recognition, and accelerates the rapid and sustainable development of real-time variable-rate fertilization technology for precision agriculture. In China, crop phenotypic monitoring technology and real-time variable-rate fertilization technology (based on agricultural RS) is gradually shifting from being reliant on the experience of other countries to a new stage more tailored to the Chinese situation. There have been a series of creative achievements in research and application that have seen China establish itself as a world-leader in crop phenotypic monitoring and precision variable-rate fertilization technology research. However, compared with other developed countries, several challenges and knowledge gaps remain in some key core technologies.

Crop models based on RS technology are recognized in modern agricultural research as an important tool that helps transform agricultural production processes to be visualization, and from the theoretical to practical levels. There is, however, an urgent need to convert significant amounts of observational data into effective information for agricultural monitoring. It is also necessary to construct analytical methods and decision-making models for data acquisition, correction, and fusion throughout the whole growth period of a crop. Focuses on the research of ‘satellite-vehicle-ground’ multi-platforms collaborative synergy with precision acquisition, decision-making, and fertilization technologies are also required to improve independent innovation and technical reserve capacity. Ultimately, achieving these targets will promote the transformation of theoretical research into practice, and enhance the ability of agricultural information technology to drive developments in the agricultural industries. In addition, real-time variable-rate fertilization technology based on crop phenotypic information is an important route for realizing intelligent precision agriculture, and this will likely play a significant role in promoting China’s future agricultural modernization, sustainable development.

## Data Availability

Original procedure and data is available on request.

## References

[1] Liu BH, Chen XP, Cui ZL (2015). Research advance in yield potential and yield gap of three major cereal crops. Chin J Eco-Agric.

[2] Zhenhua X, Caijuan G, Wenqi M (2011). Study on the relationship among yield, nutrient efficiency and benefits of grain crop in typical regional areas of China. Chin Agric Sci Bull.

[3] Yuan J, Liu CL, Li YM (2010). Gaussian processes based bivariate control parameters optimization of variable-rate granular fertilizer applicator. Comput Electron Agric.

[4] Guo JX, Kong K, Xie KL (2016). Effects of nutrient management on yield and nitrogen use efficiency of direct seeding rice. Acta Agron Sin.

[5] Bierman PM, Rosen CJ, Venterea RT (2012). Survey of nitrogen fertilizer use on corn in Minnesota. Agric Syst.

[6] Yu H, Liu D, Chen G (2010). A neural network ensemble method for precision fertilization modeling. Math Comput Model.

[7] Zhao C (2009). Research and practice of precision agriculture.

[8] Yuan J, Liu QH, Liu XM (2014). Granular multi-flows fertilization process simulation and tube structure optimization in nutrient proportion of variable rate fertilization. Trans Chin Soc Agric Mach.

[9] Gu Y, Yuan J, Liu C (2011). FIS-based method to generate bivariate control parameters regulation sequence for fertilization. Trans Chin Soc Agric Eng.

[10] Li R, Ding Y, Yu H (2017). Construction and application of the electronic prescription map system of variable rate fertilization in Southern Jiangsu. J China Agric Univ.

[11] Zaman QU, Esau TJ, Schumann AW (2011). Development of prototype automated variable rate sprayer for real-time spot-application of agrochemicals in wild blueberry fields. Comput Electron Agric.

[12] Tang H, Wang J, Xu C (2019). Research progress analysis on key technology of chemical fertilizer reduction and efficiency increase. Trans Chin Soc Agric Mach.

[13] Raun WR, Solie JB, Martin KL (2005). Growth stage, development, and apatial variability in corn evaluated using optical sensor readings. J Plant Nutr.

[14] Chen J, Tang L, Liu XJ (2011). Modeling plant nitrogen uptake and grain protein accumulation in rice. Sci Agric Sin.

[15] Xue Z, Gao H, Liu J (2011). Monitoring growth and grain yield of wheat in fields with different soil fertility levels and different fertilizer application using spectral reflectance technique. J Triticeae Crops.

[16] Maleki MR, Mouazen AM, Ramon H (2007). Multiplicative scatter correction during on-line measurement with near infrared spectroscopy. Biosyst Eng.

[17] Zhu Y, Zhu Y, Huang Y (2010). Assimilation technique of remote sensing information and rice growth model based on particle swarm optimization. J Remote Sens.

[18] Ma Y, Song X, Quan B (2015). Temporal and spatial variation of plant nitrogen and biomass of winter wheat under variable rate fertilization conditions. J Triticeae Crops.

[19] Xue L, Yang L (2008). Recommendations for nitrogen fertiliser topdressing rates in rice using canopy reflectance spectra. Biosyst Eng.

[20] Tang H (2018). Progress and prospect of agricultural remote sensing research. J Agric.

[21] Chen Z, Ren J, Tang H (2016). Progress and perspectives on agricultural remote sensing research and applications in China. J Remote Sens.

[22] Haboudane D, Miller JR, Tremblay N (2002). Integrated narrow-band vegetation indices for prediction of crop chlorophyll content for application to precision agriculture. Remote Sens Environ.

[23] Lan Y, Deng X, Zeng G (2019). Advances in diagnosis of crop diseases, pests and weeds by UAV remote sensing. Smart Agric.

[24] Basso B, Ritchie JT, Cammarano D (2011). A strategic and tactical management approach to select optimal N fertilizer rates for wheat in a spatially variable field. Eur J Agron.

[25] Song X, Wang J, Huang W, Yan G (2009). Monitoring spatial variance of winter wheat growth and grain quality under variable-rate fertilization conditions by remote sensing data. Trans Chin Soc Agric Eng.

[26] Yang JN, Zhang JC, Yao X (2013). Experiments on performance of portable plant growth monitoring diagnostic instrument. Trans Chin Soc Agric Mach.

[27] Yinyan S, Man C, Xiaochan W (2018). Numerical simulation of spreading performance and distribution pattern of centrifugal variable-rate fertilizer applicator based on DEM software. Comput Electron Agric.

[28] Wang XC, Chen M, Sun GX (2015). Design and test of control system on variable fertilizer applicator for winter wheat. Trans Chin Soc Agric Eng.

[29] Yinyan S, Zhichao H, Xiaochan W (2018). Motion analysis and system response of fertilizer feed apparatus for paddy variable-Rate fertilizer spreader. Comput Electron Agric.

[30] Shi Y, Chen M, Wang XC (2018). Design and experiment of variable-rate fertilizer spreader with centrifugal distribution cover for rice paddy surface fertilization. Trans Chin Soc Agric Mach.

[31] Shi Y, Chen M, Wang X (2018). Numerical simulation and optimization of scattering performance of a conical centrifugal variable rate fertilizer spreader. Int Agric Eng J.

[32] Shi Y, Hu Z, Wang X (2018). Fertilization strategy and application model using a centrifugal variable-rate fertilizer spreader. Int J Agric Biol Eng.

[33] Liang ZZ, Yang Y, Guo Y (2015). Status and prospect of agricultural remote sensing. Trans Chin Soc Agric Mach.

[34] Zhao CJ (2014). Advances of research and application in remote sensing for agriculture. Trans Chin Soc Agric Mach.

[35] Wu Q, Qi B, Zhao TJ (2013). A tentative study on utilization of canopy hyperspectral reflectance to estimate canopy growth and seed yield in soybean. Acta Agron Sin.

[36] Maes WH, Steppe K (2019). Perspectives for remote sensing with unmanned aerial vehicles in precision agriculture. Trends Plant Sci.

[37] Liu H, Tang L, Zhang W (2009). Construction and implementation of model-based visual rice growth system. Trans Chin Soc Agric Eng.

[38] Zhang Y, Yao X, Tian YC (2010). Estimating leaf nitrogen content with near infrared reflectance spectroscopy in rice. Chin J Plant Ecol.

[39] Ni J, Wang TT, Yao X (2013). Design and experiments of multi-spectral sensor for rice and wheat growth information. Trans Chin Soc Agric Mach.

[40] Hu P (2018). High-throughput field morphological phenotyping using UAV-based proximal sensing.

[41] He ZY, Zhu Y, Li YD (2017). Study on estimation model for nitrogen nutrition index and yield on double cropping rice in southern China. J Nanjing Agric Univ.

[42] Liu XJ, Tian YC, Yao X (2012). Monitoring leaf water content based on hyperspectra in rice. Sci Agric Sin.

[43] Fu Y, Yang G, Wang J (2014). Winter wheat biomass estimation based on spectral indices, band depth analysis and partial least squares regression using hyperspectral measurements. Comput Electron Agric.

[44] Li P, Wang Q (2011). Retrieval of leaf biochemical parameters using PROSPECT Inversion: a new approach for alleviating Ill-posed problems. IEEE Trans Geosci Remote Sens.

[45] Yang G, Zhao C, Liu Q (2011). Inversion of a radiative transfer model for estimating forest LAI from multisource and multi-angular optical remote sensing data. IEEE Trans Geosci Remote Sens.

[46] Ni J, Yao X, Tian Y (2013). Design and experiments of portable apparatus for plant growthmonitoring and diagnosis. Trans Chin Soc Agric Eng.

[47] Li Y, Shu S, Chen L (2013). Photosynthetic production simulation based on plant type in rice: a review. Chin Agric Sci Bull.

[48] Raymond Hunt E, Doraiswamy PC, McMurtrey JE (2013). A visible band index for remote sensing leaf chlorophyll content at the canopy scale. Int J Appl Earth Observ Geoinf.

[49] Liang T, Xiangcheng Z, Mengying C (2012). Relationships of rice canopy PAR interception and light use efficiency to grain yield. Chin J Appl Ecol.

[50] Yebra M, Chuvieco E (2009). Linking ecological information and radiative transfer models to estimate fuel moisture content in the Mediterranean region of Spain: solving the ill-posed inverse problem. Remote Sens Environ.

[51] Nguy-Robertson AL, Peng Y, Gitelson AA (2014). Estimating green LAI in four crops: potential of determining optimal spectral bands for a universal algorithm. Agric For Meteorol.

[52] Li Y, Zhu XC, Tang L (2011). Simulation of canopy photosynthetic production based on plant type in rice. Acta Agron Sin.

[53] Yanda L, Liang T, Yuping Z (2010). Spatiotemporal distribution of photosynthetically active radiation in rice canopy. Chin J Appl Ecol.

[54] Li W, Wang J, Zhao C (2008). A model for predicting protein content in winter wheat grain based on Land-Sat TM image and nitrogen accumulation. J Remote Sens.

[55] Tang L, Li YD, Zhang YP (2011). Simulation of canopy light distribution and application in rice. Chin J Rice Sci.

[56] Zhao C, Wang J, Huang W (2010). Spectral indices sensitively discriminating wheat genotypes of different canopy architectures. Precis Agric.

[57] Tian YC, Yang J, Yao X (2009). Quantitative relationship between hyper-spectral red edge position and canopy leaf nitrogen concentration in rice. Acta Agron Sin.

[58] He B, Quan X, Xing M (2013). Retrieval of leaf area index in alpine wetlands using a two-layer canopy reflectance model. Int J Appl Earth Obser Geoinf.

[59] Li W, Li H, Wang J (2010). A study on classification and monitoring of winter wheat growth status by Landsat/TM image. J Triticeae Crops.

[60] Dongqin Z, Yan Z, Jie Y (2009). C/N content ratio of rice leaf monitoring based on canopy hyperspectral parameters. Trans Chin Soc Agric Eng.

[61] Li YD, Tang L, Zhang YP (2010). Relationship of PAR interception of canopy to leaf area and yield in rice. Sci Agric Sin.

[62] Shibayama M, Akiyama TA (1986). A spectroradiometer for field use: VI. Radiometric estimation for chlorophyll index of rice canopy. Jpn J Crop Sci.

[63] Liu L. Hyperspectral remote sensing application in precision agriculture. Beijing: Post-doctoral Dissertation of the Chinese Academy of Sciences; 2002.

[64] Cho MA, Skidmore AK (2006). A new technique for extracting the red edge position from hyperspectral data: the linear extrapolation method. Remote Sens Environ.

[65] Chen P, Haboudane D, Tremblay N (2010). New spectral indicator assessing the efficiency of crop nitrogen treatment in corn and wheat. Remote Sens Environ.

[66] Chen P, Jiulin S, Jihua W (2010). Using remote sensing technology for crop nitrogen diagnosis: status and trends. Sci Sin Inform.

[67] Chunhua X, Shaokun L, Keru W (2008). Response of canopy direction reflectance spectrum for the wheat vertical leaf distribution. Sci Agric Sin.

[68] Li H, Zhao C, Huang W (2013). Non-uniform vertical nitrogen distribution within plant canopy and its estimation by remote sensing: a review. Field Crops Res.

[69] Wang JH, Huang WJ, Lao CL (2007). Inversion of winter wheat foliage vertical distribution based on canopy reflected spectrum by partial least squares regression method. Spectrosc Spectr Anal.

[70] Wang J, Wang ZJ, Huang WJ (2004). The vertical distribution characteristic and spectral response of canopy nitrogen in different layer of winter wheat. J Remote Sens.

[71] Zhang DY, Wang X, Coburn C (2013). Design and experiment of ground-based agriculture-oriented multi-angle observation device. Trans Chin Soc Agric Mach.

[72] Ma J, Zhu Y, Yao X (2007). Relationship between leaf nitrogen content and fluorescence parameters in rice. Chin J Rice Sci.

[73] Wu C, Han X, Niu Z (2010). An evaluation of EO-1 hyperspectral Hyperion data for chlorophyll content and leaf area index estimation. Int J Remote Sens.

[74] Qaio X, Ma X, Zhang XC (2008). Response of coronary spectrum on chlorophyll and K information of soy. Trans Chin Soc Agric Mach.

[75] Wu C, Li W, Niu Z (2010). Nondestructive estimation of canopy chlorophyll content using Hyperion and Landsat/TM images. Int J Remote Sens.

[76] Kitchen NR, Sudduth KA, Drummond ST (2010). Ground-based canopy reflectance sensing for variable-rate nitrogen corn fertilization. Agron J.

[77] Peng S, Garcia FV, Laza RC (1996). Increased N-use efficiency using a chlorophyll meter on high-yielding irrigated rice. Field Crops Res.

[78] Scharf PC, Lory JA (2002). Calibrating corn color from aerial photographs to predict sidedress nitrogen need. Agron J.

[79] Lukina EV, Freeman KW, Wynn KJ (2001). Nitrogen fertilization optimization algorithm based on in-season estimates of yield and plant nitrogen uptake. J Plant Nutr.

[80] Chen LP (2003). Theoretical and experimental studies on variable-rate fertilization in precision farming.

[81] A’ning J, Wenjiang H, Chunjiang Z (2007). Effects of variable nitrogen application based on characteristics of canopy light reflectance in wheat. Sci Agric Sin.

[82] Guo Q, Yang W, Wu F (2018). High-throughput crop phenotyping: accelerators for development of breeding and precision agriculture. Bull Chin Acad Sci.

[83] Sankaran S, Khot LR, Espinoza CZ (2015). Low-altitude, high-resolution aerial imaging systems for row and field crop phenotyping: a review. Eur J Agron.

[84] Boegh E, Soegaard H, Broge N (2002). Airborne multispectral data for quantifying leaf area index, nitrogen concentration, and photosynthetic efficiency in agriculture. Remote Sens Environ.

[85] Deery DM, Rebetzke GJ, Jimenez-Berni JA (2016). Methodology for high-throughput field phenotyping of canopy temperature using airborne thermography. Front Plant Sci.

[86] Rajan N, Maas SJ (2009). Mapping crop ground cover using airborne multispectral digital imagery. Precis Agric.

[87] Chapman SC, Merz T, Chan A (2014). Pheno-copter: a low-altitude, autonomous remote-sensing robotic helicopter for high-throughput field-based phenotyping. Agronomy.

[88] Jonckheere I, Fleck S, Nackaerts K (2004). Review of methods for in situ leaf area index determination: Part I. Theories, sensors and hemispherical photography. Agric For Meteorol.

[89] Krienke B, Ferguson RB, Schlemmer M, Holland K (2017). Using an unmanned aerial vehicle to evaluate nitrogen variability and height effect with an active crop canopy sensor. Precis Agric.

[90] Liu C, Wang Z, Chen Z (2018). Nitrogen monitoring of winter wheat based on unmanned aerial vehicle remote sensing image. Trans Chin Soc Agric Mach.

[91] Zheng H, Cheng T, Li D (2018). Combining unmanned aerial vehicle (UAV)-based multispectral imagery and ground-based hyperspectral data for plant nitrogen concentration estimation in rice. Front Plant Sci.

[92] Konishi N, Shiga H, Asaka D (2000). The application of remote sensing technology in high quality rice production. Measurement.

[93] Araus JL, Cairns JE (2014). Field high-throughput phenotyping: the new crop breeding frontier. Trends Plant Sci.

[94] Duan T, Chapman SC, Holland E (2016). Dynamic quantification of canopy structure to characterize early plant vigour in wheat genotypes. J Exp Bot.

[95] Hu P, Chapman SC, Wang X (2018). Estimation of plant height using a high throughput phenotyping platform based on unmanned aerial vehicle and self-calibration: Example for sorghum breeding. Eur J Agron.

[96] Busemeyer L, Mentrup D, Möller K (2013). BreedVision-a multi-sensor platform for non-destructive field-based phenotyping in plant breeding. Sensors.

[97] Sharma LK, Bu H, Franzen DW (2016). Use of corn height measured with an acoustic sensor improves yield estimation with ground based active optical sensors. Comput Electron Agric.

[98] Tian M, Ban S, Chang Q (2016). Use of hyperspectral images from UAV-based imaging spectroradiometer to estimate cotton leaf area index. Trans Chin Soc Agric Eng.

[99] Rebetzke GJ, Jimenez-Berni JA, Bovill WD (2016). High-throughput phenotyping technologies allow accurate selection of stay-green. J Exp Bot.

[100] Sharma B, Ritchie GL (2015). High-throughput phenotyping of cotton in multiple irrigation environments. Crop Sci.

[101] Jiang J, Zhang Z, Cao Q (2019). Use of a digital camera mounted on a consumer-grade unmanned aerial vehicle to monitor the growth status of wheat. J Nanjing Agric Univ.

[102] Chattha HS, Zaman QU, Chang YK (2014). Variable rate spreader for real-time spot-application of granular fertilizer in wild blueberry. Comput Electron Agric.

[103] Dammer KH, Wollny J, Giebel A (2008). Estimation of the Leaf Area Index in cereal crops for variable rate fungicide spraying. Eur J Agron.

[104] Ehlert D, Adamek R, Horn HJ (2009). Laser rangefinder-based measuring of crop biomass under field conditions. Precis Agric.

[105] Benitez Ramirez M. Monitoring nitrogen levels in the cotton canopy using real-time active-illumination spectral sensing. University of Tennessee-Knoxville. 2010.

[106] Lee W, Searcy SW. Multispectral sensor for detecting nitrogen in corn plants. In: 2000 ASAE annual international meeting sponsored by ASAE, July 9–12, 2000.

[107] Debuisson S, Germain C, Garcia O, Panigai L, et al. Using multiplex® and greenseeker^™^ to manage spatial variation of vine vigor in champagne.

[108] Yuan Y, Li S, Fang X (2013). Decision support system of N, P and K ratio fertilization. Trans Chin Soc Agric Mach.

[109] Qiao X (2008). Research of soybean nutrition information diagnosis based on spectrum technology and system of variable rate fertilizer.

[110] Zhang R (2012). The research on key technology of intelligence variable rate fertilization.

